# Two successful projects from EIT RawMaterials on digitalization and responsible mineral exploration

**DOI:** 10.1007/s13563-021-00252-9

**Published:** 2021-02-16

**Authors:** Per Storm, Mia Smeds

**Affiliations:** 1 EIT RawMaterials Innovation Hub North, Stockholm, Sweden; 2 EIT RawMaterials Innovation Hub North, Helsingfors, Finland

EIT RawMaterials, initiated and funded by the EIT (European Institute of Innovation and Technology), a body of the European Union, is the largest consortium in the raw materials sector worldwide. Its vision is to develop raw materials into a major strength for Europe. Its mission is to enable sustainable competitiveness of the European minerals, metals, and materials sector along the value chain by driving innovation, education, and entrepreneurship.

EIT RawMaterials unites more than 120 core and associate partners and 180+ project partners from leading industries, universities, and research institutions from more than 20 EU countries. Partners of EIT RawMaterials are active across the entire raw materials value chain, from exploration, mining, and mineral processing to substitution, recycling, and circular economy. They collaborate on finding new, innovative solutions to secure the supplies and improve the raw materials sector in Europe.

Two successful projects in the Nordic countries are NITREM, where scientist and industry have developed new technology for removing nitrogen, and MULSEDRO, where the consortium has created a unique multi-sensor drone system for fast and inexpensive mineral exploration.

## New technology for removing nitrogen from mine water

Nitrogen impacts on the environment from mining operations are reduced through bioreactor technology in combination with a geomorphic landscape design

An effective method to purify water leaking from mine waste deposits from nitrogen has been developed in the innovation project NITREM (www.nitrem.eu) supported by EIT RawMaterials (www.eitrawmaterials.eu). The technology development has been driven by EU’s Water Framework Directive and will enable the industry to meet current and future discharge requirements. The outcome is a low-cost and low-maintenance technology ready for market introduction and customer testing.

The bioreactor technology was previously evaluated in a pilot-scale system at EIT RawMaterials partner LKAB’s Kiruna mine (https://www.lkab.com/en/about-lkab/from-mine-to-port/mining/) in northern Sweden, and is currently being tested in a full-scale operation at the same mine. The Kiruna mine is the largest and most modern underground iron ore mine in the world.

### Full-scale implementation of the innovative bioreactor technology

“Our primary achievement is the full-scale implementation of the innovative bioreactor technology in an operational mining environment. We have been able to remove most of the nitrogen for extended periods at low temperature,” says Dr Roger Herbert, Project Coordinator at Uppsala University.

The NITREM innovation project has been running for three years and is developing a service that combines the geomorphic design of waste rock deposits, a water collection system, and a bioreactor technology to reduce the nitrogen content in the leachate from waste rock. A pre-study has investigated the implementation of the NITREM service in a new depositional area that will receive approximately 200 Mt of waste rock.

EIT RawMaterials partner LKAB, an international high-tech mining and mineral group in Sweden, has been working for a long time to find methods for purifying process water and removing nitrogen from more diffuse sources such as waste rock deposits. Every night when LKAB detonates explosives in the mine, ammonium nitrate–based explosives are used. Residual nitrate remains after blasting and is ultimately left to leach from waste rock piles.

“The nitrogen thus ends up directly in the mining and process water or ends up in deposits where it slowly causes diffuse emissions to surrounding lakes and streams. It affects the environment negatively, because nitrogen promotes the growth of algae and organisms and leads to eutrophication,” says Mattias Ylipää, Senior Research Engineer at LKAB.

### Seventy-seven percent of the nitrogen removed from the water

The NITREM service is applicable and targeted to the mining and rock quarry industries, and includes a geomorphic landform design for waste rock dumps, which provides benefits for bioreactor optimization and addresses the environmental and cultural sensitivity often linked with mining and quarry sites.

In the summer of 2018, the first bioreactor within the EIT RawMaterials project was built near a waste rock deposit in Kiruna, Sweden. Now, two years later, water sampling shows that the bioreactor is an effective method of purifying water from nitrogen.

“During a 165-day sampling period, 77% of the nitrogen was removed from the water, which may be considered good given that this is a passive system that takes care of itself. Nitrate concentrations in the incoming water were around 70 milligrams per liter and in the outgoing water below 10 milligrams per liter,” says Mattias Ylipää.

LKAB is now reviewing the possibilities of building bioreactors next to the planned southern waste rock deposit at LKAB’s industrial area in Norrbotten in Sweden.

“The benefits of bioreactors are many. It is relatively cheap to build this type of treatment plant and it basically takes care of itself. It seems to be a good method for purifying nitrogen-rich leachate,” says Susanne Larsson, Project Manager at LKAB.

### The landforms created by waste rock surpass standards

Given the environmental and cultural sensitivity of areas surrounding mine and quarry sites, it is important that the aesthetic, socioeconomic, and environmental impacts of waste rock deposits are minimized.

The Sámi are the recognized indigenous peoples of the Arctic parts of Sweden, and a requirement for mining permits in this region is that waste rock dumps are restored with an ecologically appropriate vegetation cover that enables the grazing by reindeer on these sites after they have been returned to society.

The idea in this project is to provide a service where the landforms created by waste rock will achieve and surpass standards set by companies and the authorities, while the bioreactor technology minimizes the environmental impact of nitrogen releases.

The project consortium consists of Uppsala University, WSP Sverige AB, Cedervall Arkitekter, Agencia Estatal Consejo Superior de Investigaciones Cientificas, Boliden Mineral, LKAB, LTU Business, and the Swedish University of Agricultural Sciences. All organisations are partners in the largest consortium in the raw materials sector worldwide EIT RawMaterials.

## Multi-sensor drones for fast and inexpensive mineral exploration

In another successful project, supported by EIT RawMaterials, the project members have developed a multi-sensor drone system for fast and inexpensive surveying for mineral exploration. This fixed-wing drone system is a fruitful result of the project MULSEDRO: Multi-sensor drones for geology mapping (https://eitrawmaterials.eu/project/mulsedro/, https://eng.geus.dk/products-services-facilities/publications/geus-bulletin/bulletin-43/201943-03-02).

The Finish company Radai Ltd. (https://radai.fi/2020/10/16/mulsedro-project/) has, together with other partners in this upscaling project, developed an automated system that can cover large areas in a short time. The multi-sensor drone system has proven to give reliable results under Finish winter conditions and in mountainous and arctic regions such as Greenland (https://www.isaaffik.org/mulsedro-multi-sensor-drones).

“Noise disturbances are low, and the areas that can be surveyed with drones without causing any environmental harm is comparable with small conventional helicopter-borne surveys. In addition, drone surveys can be acquired at shallower altitudes and provide information in very high resolution,” says Dr Björn Heincke, Project Coordinator of MULSEDRO.

### Rental service of the drone system

Radai Ltd. has been able to significantly scale up its business thanks to the development of the new drone system within the MULSEDRO project. Next to its own surveying activities, it established a rental service of the drone system for industrial partners and exploration companies that do not wish to invest in the technology but require the benefits of the technology. Hence, making it affordable and accessible to smaller exploration companies as well.

“The development made in the MULSEDRO project has had a major impact on our business. Today, our drone system is presumably the only light-weight fixed-wing system that can gather simultaneously high-resolution multi-spectral and magnetic data. This data is highly relevant when characterizing mineral potentials of exploration areas,” says Ari Saartenoja, CEO at Radai Ltd.

As part of the EIT Crisis Response Initiative (https://eit.europa.eu/our-activities/covid-19-response/eit-crisis-response-initiative), EIT RawMaterials supports Radai Ltd. with Booster funding to accelerate business in the post-crisis economy. The EIT RawMaterials Booster Call in response to the COVID-19 crisis mobilised EUR 9.8 million to provide targeted support to 60 high-impact and growth potential start-ups, scale-ups, and SMEs during the crisis.

Radai Ltd. started its business in 2014 with developing a first magnetic drone system and joined the MULSEDRO project in 2017. Since then, their system has permanently improved, and their drone surveying activities for mineral exploration customer have been growing every year. Today, Radai Ltd. offers magnetic surveys as a standard service for the mineral exploration industry and governmental organizations in Nordic countries, including the Arctic. Within the MULSEDRO project, Radai Ltd. was able to extend their system by a multi-spectral camera, which allows for acquiring magnetic and remote sensing data in parallel.

“So far, we have more than 50 successful surveys for more than ten different customers. We have covered about 1500 km^2^ and a total of 35,000 line kilometers. This would not have been possible without the development of the fixed-wing drone system done in the MULSEDRO project and without support from EIT RawMaterials,” says Ari Saartenoja.

Partners within the MULSEDRO project were, in addition to Radai Ltd., the National Geological Surveys from Finland (GTK) and Denmark and Greenland (GEUS), the German Helmholtz-Institute for Resource Technology (HZDR-HIF), the exploration technology developing company DMT GmbH & Co. KG, and the business developer LTU Business AB.

